# Association of the stress hyperglycemia ratio and clinical outcomes in patients with stroke: A systematic review and meta-analysis

**DOI:** 10.3389/fneur.2022.999536

**Published:** 2022-09-01

**Authors:** Yong-Wei Huang, Xiao-Shuang Yin, Zong-Ping Li

**Affiliations:** ^1^Department of Neurosurgery, School of Medicine, Mianyang Central Hospital, University of Electronic Science and Technology of China, Mianyang, China; ^2^Department of Immunology, School of Medicine, Mianyang Central Hospital, University of Electronic Science and Technology of China, Mianyang, China

**Keywords:** stroke, stress hyperglycemia, stress hyperglycemia ratio, ratio of glucose to glycated hemoglobin, clinical outcome, meta-analysis

## Abstract

**Objective:**

Stress hyperglycemia (SH) is common in patients with acute diseases, such as stroke and myocardial infarction. Stress hyperglycemia ratio (SHR) is calculated by glucose/glycated hemoglobin and has been widely used for evaluating SH. But whether SHR is associated with clinical outcomes in stroke patients remains unclear so far. Although many studies have shown that higher SHR means poor outcomes, there is still no absolute evidence that SHR plays a critical role in stroke patients. Hence, we performed a systematic review and meta-analysis aiming to investigate the association between SHR and clinical outcomes in stroke patients.

**Methods:**

We performed a comprehensive literature search of the PubMed, Embase, Cochrane Library databases, Clinicaltrials.gov, and WHO-ICTRP. Following the Preferred Reporting Items for Systematic Reviews and Meta-Analyses (PRISMA), we performed our study. The Newcastle-Ottawa Scale (NOS) tool was used to examine the potential bias of included studies. The endpoints including poor outcome, mortality, neurological deficit, hemorrhagic transformation (HT), and infectious complications were statistically analyzed.

**Results:**

Sixteen retrospective studies met the eligibility criteria, and a number of 183,588 patients were included. Our meta-analysis demonstrated a significant increase in the incidence of poor outcome, according to assessment by the modified Rankin Scale (mRS) ≥ 3 points [odds ratio (OR) 2.53, 95% confidence interval (CI) 1.99–3.22, *P* < 0.00001, *I*^2^ = 68%], mortality (OR 1.96, 95% CI 1.58–2.44, *P* < 0.00001, *I*^2^ = 61%), neurological deficit (OR 1.99, 95% CI 1.47–2.70, *P* < 0.00001, *I*^2^ = 75%), hemorrhagic transformation (HT) (OR 3.70, 95% CI 2.69–5.08, *P* < 0.00001, *I*^2^ = 0%), and infectious complications [(Pneumonia) OR 2.06, 95% CI 1.57–2.72, *P* < 0.00001, *I*^2^ = 24%; (Urinary tract infection) OR 2.53, 95% CI 1.45–4.42, *P* = 0.001, *I*^2^ = 57%] in stroke patients with higher SHR. However, no significant influence was observed for recanalization rate (OR 0.86, 95% CI 0.54–1.38, *P* = 0.53, *I*^2^ = 0%).

**Conclusion:**

With or without diabetes, no matter whether undergoing intravenous thrombolysis or mechanical thrombectomy, higher SHR significantly increased the occurrence of poor outcomes, mortality, neurological deficit, HT, and infectious complications. The recanalization rate was not statistically significant between the two groups. More attention must be paid in clinical practice to SH. Future investigation should focus on the diagnostic value of SHR and the early control of hyperglycemia. Meanwhile, whether SHR could become a novel and promising target for early intervention is worthy of attention in further research. Besides, the influence of the dynamic change of glucose-to-HbA1c ratio, namely SHR, on intracerebral hemorrhage outcomes requires further investigation in future research. Although no randomized double-blind studies have been conducted, the available massive sample studies reflect the actual situation in the clinic and assist clinical decision makers.

**Systematic review registration:**

https://www.crd.york.ac.uk/prospero/, identifier: CRD42022345587.

## Introduction

Stroke, including ischemic and hemorrhagic, is a pervasive type of acute cerebrovascular disease among which hemorrhagic stroke is the second most common stroke sub-type leading to the highest morbidity and mortality (1, 2). Even though treatment for stroke patients is timely and effective now, the earlier intervention of the risk factors for adverse results is still vital to optimize outcomes. In the past 40 years, the stroke burden in China has increased without a stop, and in the recent past 7 years (from 2013 to 2019), the prevalence of stroke in China has continued to increase (3). In 2017, stroke was the leading cause of death, years of life lost, and disability-adjusted life years at the national level in China (4). An investigation involving 480,687 adults aged ≥ 20 years showed that the age-standardized prevalence and incidence rate of stroke were 1,114.8/100,000/year and 246.8/100,000/year, respectively (5). Therefore, the prevention and treatment of stroke still have a long way to go.

Stress hyperglycemia (SH), known as transient hyperglycemia secondary to neurohormonal disorders and inflammation reaction (6), is a common manifestation found in patients with myocardial infarction, stroke, and other critical illnesses (6–9). Stress hyperglycemia ratio (SHR) was first applied for assessing SH by Roberts et al. (10). Because of the stability of glycosylated hemoglobin (HbA1c) in patients with diabetes over the previous 8–12 weeks, SHR was defined as the admission glucose concentration/estimated average glucose (eAG) concentration (10, 11). However, due to discrepancies between eAG and average blood glucose, some scholars pointed out that eAG should be carefully used for clinical practice. Another definition of SHR using the ratio of glucose to HbA1c was more practical and widely applied.

SH is associated with the severity of stroke (12, 13) and poor outcomes, especially in patients without diabetes mellitus (7). Nevertheless, the association between SH and the outcomes of patients with diabetes mellitus is controversial, not only for stroke patients but also for some other critical illnesses (12, 14, 15). A study concentrating on acute ischemic stroke patients with diabetes showed that SHR could be a better predictor for the severity and poor outcome of stroke (16). But owing to its characteristic of a single-center and small sample study, the limitation of the results was obvious. Because admission glucose could be influenced by the diabetic status and the food. Therefore, fasting blood glucose (FBG) rather than random or admission glucose could be a more reliable marker, as previously suggested (17).

Many studies evaluating the association between SHR and clinical outcomes in patients with stroke have been performed in recent years (18–33). But whether SHR is associated with clinical outcomes in stroke patients remains unclear. So far, no systematic reviews and meta-analyses have been reported concerning the SHR and clinical outcomes in patients with stroke and there is still no absolute evidence that SHR plays a critical role in stroke patients. Hence, we performed a systematic review and meta-analysis aiming to investigate the association between SHR and clinical outcomes in stroke patients. Herein, we performed the first meta-analysis based on the available studies to determine the followings: (1) the relationship between SHR and clinical outcomes during the follow-up in stroke patients; (2) the influence on recanalization rate in patients accepting mechanical thrombectomy or intravenous thrombolysis.

## Methods

### Aims and PICO statement

This study was performed by the Preferred Reporting Items for Systematic Reviews and Meta-Analyses (PRISMA) (34) and was registered with PROSPERO (CRD42022345587) (35). The detailed information is presented in Supplementary Table S1. And the PICO statements were as follows: (1) **Population**: Stroke patients with or without diabetes. (2) **Intervention**: Mechanical thrombectomy or intravenous thrombolysis or neither. (3) **Comparisons**: Relative low SHR *vs*. relative high SHR (based on different groupings, if there are three groups, we defined the first group as low SHR and the rest of two groups as high SHR. Similarly, if there are four groups, the first two groups are low SHR and the remaining two groups are high SHR. (4) **Outcomes**: We defined poor outcome as the mRS ≥ 3 points at follow-up. Symptomatic intracerebral hemorrhage (SICH) and intracerebral hemorrhage (ICH) were regarded as HT. Besides, mortality, neurological deficit, recanalization rate, and infectious complications were also extracted during the follow-up.

### Literature search strategy

We performed a comprehensive literature search of the PubMed, Embase, and Cochrane Library databases. Two reviewers (Huang YW and Yin XS) systematically screened the electronic databases for the appropriate articles that were published from inception to the end of July 2022. Meanwhile, the clinical trials registry centers, including *clinicaltrials.gov* and *WHO-ICTRP*, were also screened for possible findings. The following search strategy was applied: (“stroke” [all fields]) AND (“stress hyperglycemia” [all fields]) for the above databases and the clinical trials registry centers. The detailed search strategy is presented in Supplementary Table S2.

### Inclusion and exclusion criteria

All potential studies were appraised independently with regard to the inclusion and exclusion criteria by two reviewers (Huang YW and Yin XS). The investigators selected studies that met all the following criteria: (1) types of publication: articles published in peer-reviewed medical journals; (2) types of participants: stroke patients with complete data on FBG and HbA1c upon admission; (3) types of comparison: relative low SHR *vs*. relative high SHR; (4) types of outcome measure: poor outcome, according to assessment by the mRS ≥ 3 points; mortality; neurological deficit; HT; infectious complications (pneumonia and urinary tract infection) and recanalization rate.

Case reports, reviews, notes, meta-analyses, editorials, letters to the editor, commentaries, conference abstracts, and non-English studies were excluded.

### Data extraction

Two reviewers independently extracted data using the same standardized tables. The following information was extracted from the included studies: (1) basic characteristics: study ID (year of publication + first author name), country, study design, and number of participants; (2) participant characteristics: rate of male, type of stroke, operation, primary endpoint, secondary endpoint, and clinical follow-up; (3) data on outcomes of interest, etc.

### Risk of bias assessment

The Newcastle-Ottawa Scale (NOS) tool (36) was applied to appraise the potential risk of bias (RoB) in included studies. The approach based on NOS included three parts (each part three points): (1) the selection of studies; (2) the comparability of studies; (3) the assessment of exposure/outcome. Each study might be appraised on up to 9 points. More than 6 scores were considered to indicate the high quality of the study. The assessment was performed independently by three reviewers (Huang YW, Yin XS, and Li ZP). Any differences were resolved in a group investigator discussion if required.

### Statistical analysis

We calculated odds ratios (ORs) and their corresponding 95% confidence interval (CIs) when comparing the different endpoints of high SHR and low SHR among stroke patients. Considering clinical heterogeneity, we used DerSimonian and Laird random-effects model to perform the meta-analyses (37). *P*-value < 0.05 was considered statistically significant. The heterogeneity between studies was appraised by the Cochrane Q test (*P* < 0.1 or *I*^2^ > 50% was considered to represent significant heterogeneity) (38). Specific data of the high SHR and low SHR groups were extracted from the studies based on our definition of high SHR and low SHR. The possibility of publication bias was assessed by the analysis of the funnel plot. All statistical analyses were conducted with the Review Manager software (version 5.3.0; https://training.cochrane.org/online-learning/core-softwarecochrane-reviews/revman).

## Results

**Figure 1:**
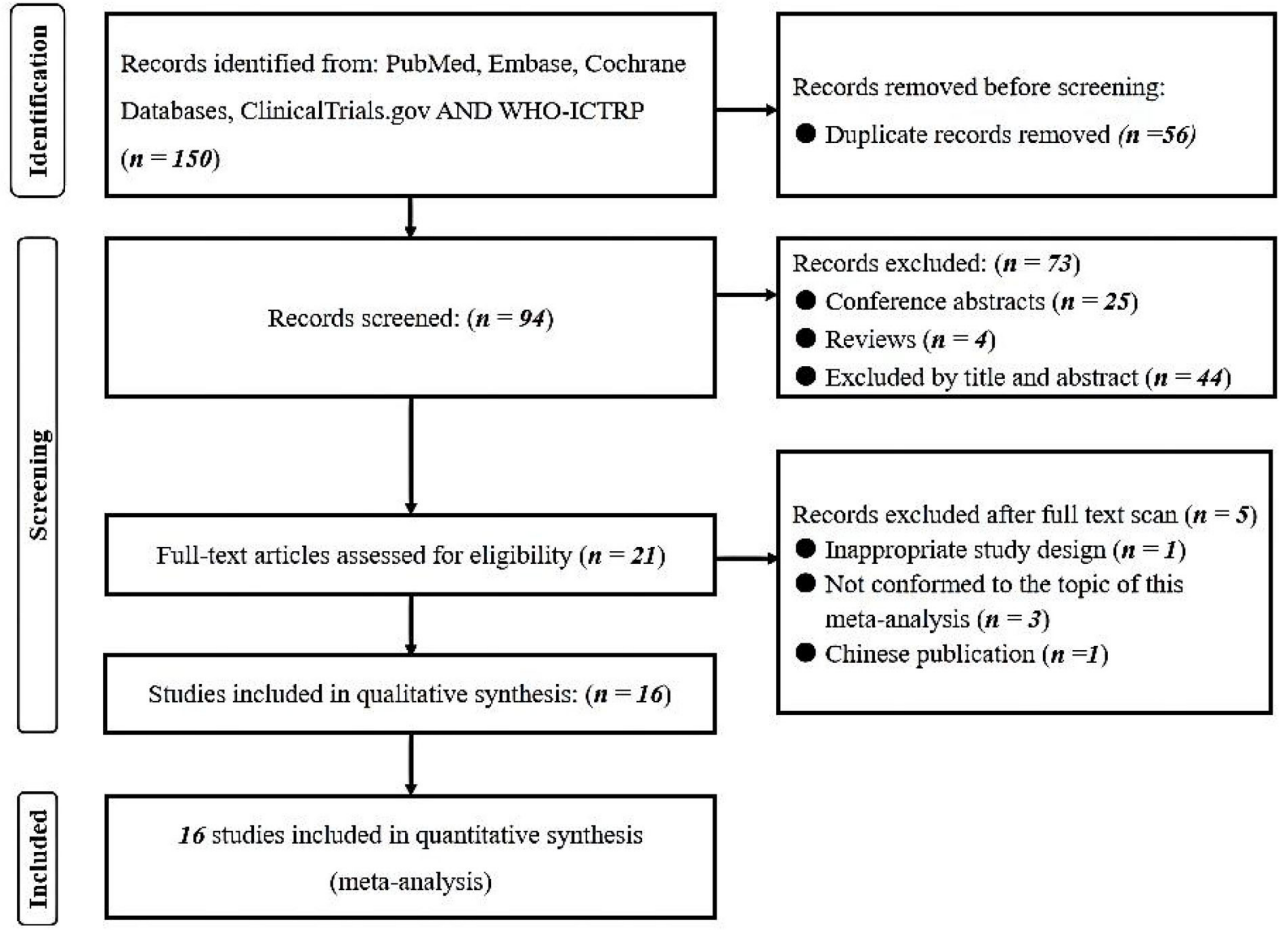
PRISMA flowchart of included studies.

A comprehensive literature search of the PubMed, Embase, Cochrane Library databases, clinicaltrials.gov and WHO-ICTRP was performed. A total of 150 records were identified. Twenty-one articles underwent a full-text evaluation, five of which were excluded (one for inappropriate study design, three for inappropriate topic, and one for Chinese publication), leaving altogether sixteen studies in this systematic review and meta-analysis (18–33). The flowchart based on PRISMA is summarized in Figure 1. We identified five multi-center retrospective and 11 single-center retrospective studies. A number of 183,588 patients were included and the results are summarized in Table 1.

**Table 1:** Baseline characteristic description of the included studies.

**References**	**Country**	**Study design**	**Participants**	**Male-%**	**Type of stroke**	**Operation**	**Primary endpoint**	**Secondary endpoint**	**Clinical follow-up**
Chen et al. (18)	China	Retrospectively single-center	160	67.5	Ischemic stroke	Mechanical thrombectomy	Poor outcome	—	3 months
Wang et al. (19)	China	Retrospectively single-center	321	61.1	Ischemic stroke	Mechanical thrombectomy	Mortality	SICH Infectious complications	3 months
Zhu et al. (20)	China	Retrospectively multi-center	999	64.4	Ischemic stroke	—	Mortality	Stroke recurrence	12 months
Li et al. (21)	China	Retrospectively multi-center	8,622	62.8	Ischemic stroke	—	Mortality	Neurological deficit	Discharge 3 months
Merlino et al. (22)	Italy	Retrospectively single-center	414	53.4	Ischemic stroke	Intravenous thrombolysis	Poor outcome Mortality SICH	Neurological deficit in-hospital mortality ICH	3months
Merlino et al. (23)	Italy	Retrospectively single-center	204	49.0	Ischemic stroke	Mechanical thrombectomy	Poor outcome Mortality SICH	Neurological deficit in-hospital mortality ICH	3months
Roberts et al. (24)	Australia	Retrospectively single-center	300	53.0	Ischemic stroke	—	Poor outcome	—	discharge
Shen et al. (25)	China	Retrospectively single-center	341	70.7	Ischemic stroke	Intravenous thrombolysis	Poor outcome	Mortality Neurological deficit SICH HT	3months
Yuan et al. (26)	China	Retrospectively single-center	572	68.4	Ischemic stroke	—	HT	—	—
Cai et al. (27)	China	Retrospectively single-center	846	61.7	Ischemic stroke/hemorrhagic stroke	—	Poor outcome Mortality	Infectious complications	3 months 12 months
Chen et al. (28)	China	Retrospectively single-center	230	62.2	Ischemic stroke	Intravenous thrombolysis	Poor outcome Neurological deficit	Mortality	3 months
Chu et al. (29)	China	Retrospectively multi-center	313	72.5	Hemorrhagic stroke	—	Poor outcome	Neurological deficit Mortality	3 months
Li et al. (30)	China	Retrospectively multi-center	586	70.3	Hemorrhagic stroke	—	Poor outcome	—	3 months
Merlino et al. (31)	Italy	Retrospectively single-center	501	53.9	Ischemic stroke	Intravenous thrombolysis	Poor outcome Mortality SICH	Neurological deficit in-hospital mortality ICH	3 months
Mi et al. (32)	China	Retrospectively multi-center	168,381	57.0	Ischemic stroke	—	Mortality	—	12 months
Wang et al. (33)	China	Retrospectively single-center	798	64.2	Ischemic stroke	Intravenous thrombolysis	Poor outcome	Neurological deficit in-hospital mortality SICH	discharge

### Heterogeneity

According to the results of the studies, a moderate statistical heterogeneity was found with poor outcome (*P* = 0.0008 for Cochran Q, *I*^2^ = 68%), mortality (*P* = 0.004 for Cochran Q, *I*^2^ = 61%), neurological deficit (*P* = 0.0005 for Cochran Q, *I*^2^ = 75%), infectious complications (*P* = 0.13 for Cochran Q *I*^2^ = 57%). Therefore, a random-effect model was used in these endpoints. The results are summarized in Table 2.

**Table 2:** Heterogeneity and meta-analysis of included studies.

**Items**	**Trials, *n***	**Results**		
		**OR (95% CI)**	***p*** **-value**	**Heterogeneity (I^2^, *p* for Cochran Q)**
Poor outcome	10	2.53 (1.99–3.22)	*p* < 0.00001	I^2^ = 68%, *P* = 0.0008
Mortality	11	1.96 (1.58–2.44)	*p* < 0.00001	I^2^ = 61%, *P* = 0.004
Neurological deficit	7	1.99 (1.47–2.70)	*p* < 0.00001	I^2^ = 75%, *P* = 0.0005
Hemorrhagic transformation	7	3.70 (2.69–5.08)	*p* < 0.00001	I^2^ = 0%, *P* = 0.69
Pneumonia	3	2.06 (1.57–2.72)	*p* < 0.00001	I^2^ = 24%, *P* = 0.27
Urinary tract infection	2	2.53 (1.45–4.42)	*p* = 0.001	I^2^ = 57%, *P* = 0.13
Recanalization rate	2	0.86 (0.54–2.04)	*p* = 0.53	I^2^ = 0%, *P* = 0.32

### Meta-analysis of different outcomes

**Figure 2:**
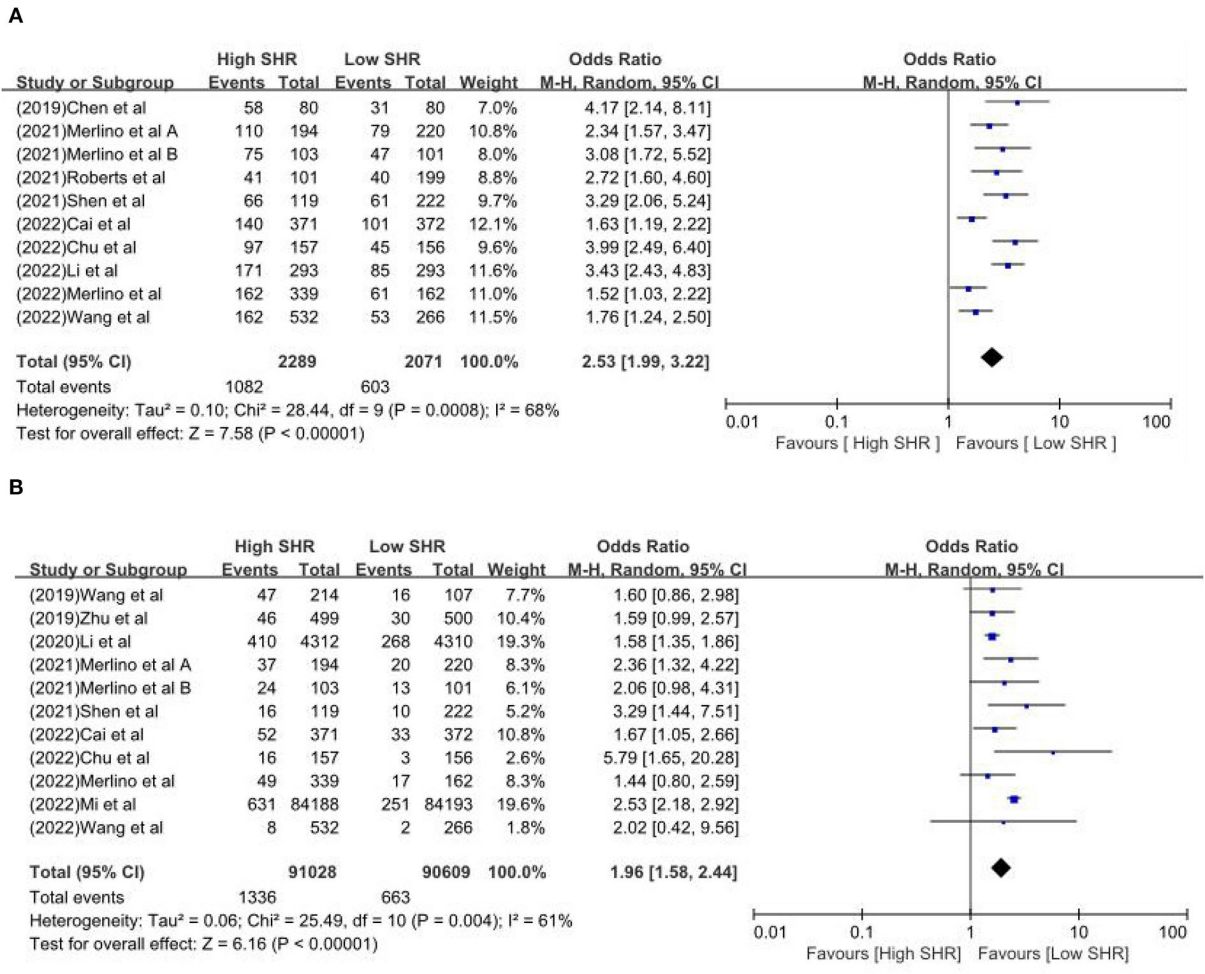
The **(A)** Poor outcome and **(B)** Mortality between high SHR and low SHR groups.

**Figure 3:**
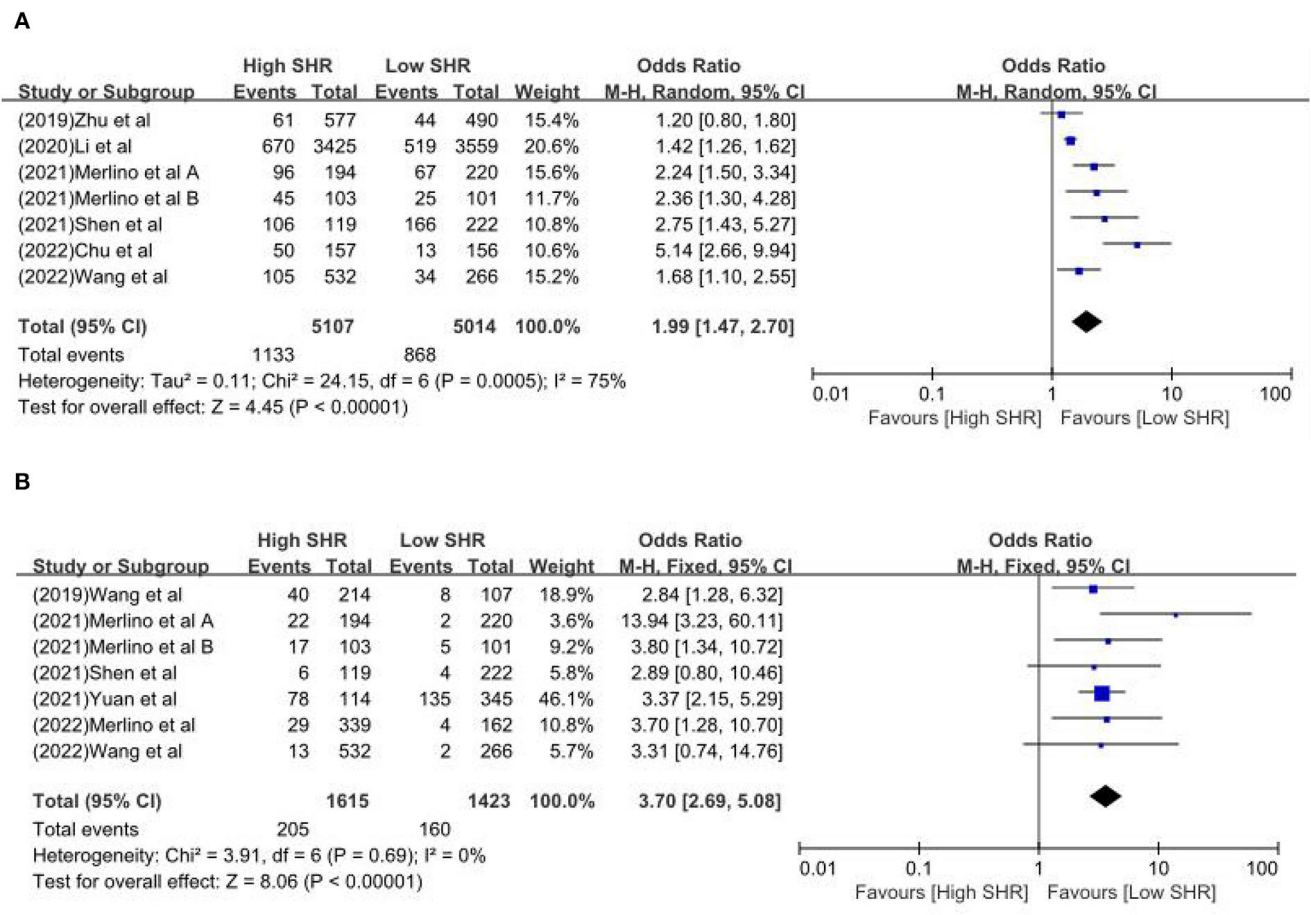
The **(A)** Neurological deficit and **(B)** hemorrhagic transformation between high SHR and low SHR groups.

**Figure 4:**
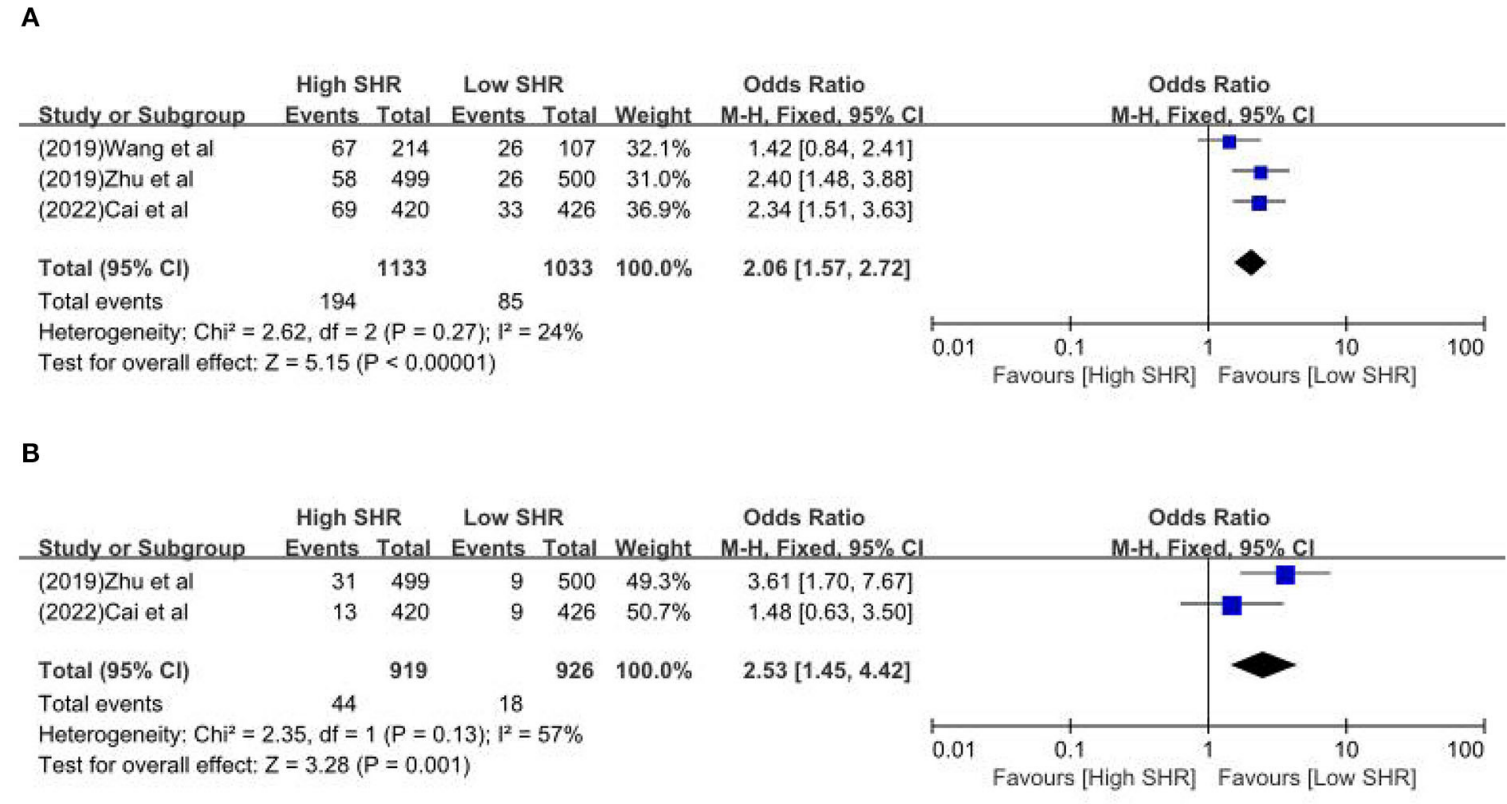
The **(A)** Pneumonia and **(B)** urinary tract infection between high SHR and low SHR groups.

**Figure 5:**
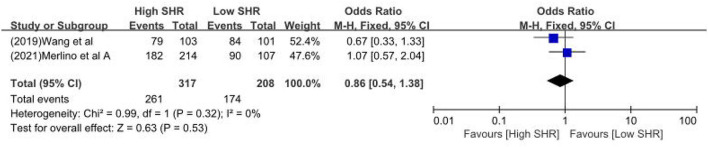
The recanalization rate between high SHR and low SHR groups.

The results are summarized in Table 2. The meta-analysis demonstrated a significant increase in the incidence of poor outcome (mRS ≥ 3 points) [odds ratio (OR) 2.53, 95% confidence interval (CI) 1.99–3.22, *P* < 0.00001, *I*^2^ = 68%; Figure 2A], mortality (OR 1.96, 95% CI 1.58–2.44, *P* < 0.00001, *I*^2^ = 61%; Figure 2B), neurological deficit (OR 1.99, 95% CI 1.47–2.70, *P* < 0.00001, *I*^2^ = 75%; Figure 3A), hemorrhagic transformation (HT) (OR 3.70, 95% CI 2.69–5.08, *P* < 0.00001, *I*^2^ = 0%; Figure 3B), and infectious complications [(Pneumonia) OR 2.06, 95% CI 1.57–2.72, *P* < 0.00001, *I*^2^ = 24%; Figure 4A; (Urinary tract infection) OR 2.53, 95% CI 1.45–4.42, *P* = 0.001, *I*^2^ = 57%; Figure 4B] in patients with higher SHR. However, no significant benefit was observed for re-canalization rate (OR 0.86, 95% CI 0.54–1.38, *P* = 0.53, *I*^2^ = 0%; Figure 5).

### Risk of bias assessment

All these studies were marked as having low levels of RoB according to the NOS tool within the following items: selection bias, detection bias, and reporting bias. All studies were retrospective and with a mean of 7.69 stars and a standard deviation (SD) of 0.98 stars. The methodological quality of the included studies is presented in Supplementary Table S3.

## Discussion

It is generally believed that the key points of SH are the activation of the hypothalamic-pituitary axis and sympatho-adrenal system causing the increases in the release of epinephrine, norepinephrine, and pro-inflammatory cytokines (TNF-α, IL-1, and IL-6) (39). The underlying mechanism of SH is as follows: first, strong inflammatory and neurohormonal responses caused increased induction of endothelial apoptosis and oxidative stress (OS) (6). In detail, activation of matrix metalloproteinase gelatinase B (MMP-9), breakdown of the blood-brain barrier (BBB), and BBB leakage increased brain edema and hemorrhage causing severe neurological deficits (40). Second, stress hormones can stimulate hepatic gluconeogenesis and inhibit glucose uptake in peripheral tissues (39). Pro-inflammatory cytokines, by upregulating expression and membrane localization of glucose transporters GLUT-1 and GLUT-3, facilitated the glucose uptake. They are used by the peripheral and central nervous systems (41). Besides, cellular glucose overload caused an increase in brain lactate production and further transformed asymptomatic tissue into symptomatic tissue (42). Third, no matter acute or chronic hyperglycemia, all play a particularly critical role in prothrombotic shift (43) and may facilitate thrombus extension (44). Fourth, SH may reflect the transient glycemic change. The glucose fluctuations exhibited a more specific triggering effect on OS (45). Finally, the degree of SH, named SHR, may reflect the severity of diseases. In patients with stroke, SHR can represent the extent of ischemic damage and cause poor clinical outcomes.

One study investigated by Chen et al. (18) demonstrated that increased SHR is strongly correlated with poor outcome at 3 months after MT for proximal artery occlusion in the anterior circulation (high SHR 72.5% vs. low SHR 38.8%). But the result was limited to being significant in non-diabetic stroke patients, not in stroke patients with diabetes. Poor glycemic control seemed to be associated with poor functional outcomes after stroke. That meant long-term glycemic stress and damage are involved in the functional prognosis of stroke, while acute hyperglycemia after stroke might be a predictor of death. Another relevant study conducted by Wang et al. (19) focused on the mortality risk, and they found that higher SHR was associated with higher mortality risk after MT in acute ischemic stroke patients (high SHR 22.0% vs. low SHR 15%). Zhu et al. (20) performed a study focusing on non-diabetic stroke patients and showed that SHR was related to an elevated risk of stroke recurrence and all-cause death. Li et al.'s (21) study found that SHR was associated with an increased risk of severe neurological deficit and mortality within 1 year in acute ischemic stroke people with and without diabetes. In 2021, two studies from Italy demonstrated that SHR is associated with worse outcomes and detrimental effects in stroke patients undergoing intravenous thrombolysis or mechanical thrombectomy (22, 23). Another two relevant studies focus on hemorrhagic stroke and demonstrated that SHR is a reliable predictor for early hematoma expansion and poor outcomes and SHR was independently correlated with worse functional outcomes at discharge and 3 months in patients with ICH (29, 30). Li et al. (30) showed that SHR was independently correlated with worse functional outcomes at discharge and 3 months in patients with ICH. Besides, SHR could be used as a simple and readily available index to predict clinical outcomes of ICH. The study of SH provides meaningful insight into optimal glucose levels among ICH patients and develops tailored glucose-lowering strategies (30). Chen et al.'s investigation suggested that SHR is expected to replace random or fasting glucose concentration as a novel generation of prognostic indicator and a potential therapeutic target (28). However, Merlino et al. (31) found that SHR was not associated with the clinical outcome of diabetic patients receiving intravenous thrombolysis for acute ischemic stroke. Mi et al. (32) conducted a massive sample and multi-center study involving 168,381 stroke patients from the Chinese Stroke Center Alliance (CSCA) database. Based on their findings, they considered that the SHR may serve as an accessory parameter for the prognosis of patients with diabetes after acute ischemic stroke, and hyperglycemia in stroke patients with diabetes mellitus is associated with a higher risk of in-hospital death. One has confirmed that SH has a certain predictive value for hemorrhagic transformation in patients with AIS (26).

SH is a common manifestation found in patients with critical illnesses, especially in stroke patients. As Li et al. (30) said, SHR was a simple and readily available index to predict clinical outcomes. In clinical practice, we need such an index that is easy to use and appraise possible clinical outcomes of stroke patients. For instance, some imaging markers, such as island sign (46) and blend sign (47) on the baseline computed tomography scan, identify the high-risk patients of hematoma expansion by this non-invasive to provide earlier clinical intervention aiming to decrease mortality and disability. In fact, the SHR is similar to this. Because of its convenience and non-invasive, SHR may be widely used for our screening of high-risk stroke patients and earlier identification of the adverse results. If further studies in the future aim to establish the prediction model or artificial intelligence algorithm for predicting the clinical outcomes of stroke patients, the SHR may serve as an important component of the associated model or algorithm. Altogether, SHR is an important prognosis factor for stroke patients and is helpful for clinicians to identify the high-risk population for stroke.

Our meta-analysis has comprehensively and systematically reviewed the currently available literature that compared different SHR in stroke patients with/without diabetes, and we obtained three major findings. First, in patients with stroke, higher SHR indicated poor outcome, mortality, neurological deficit, HT, and infectious complications. But the studies on infectious complications are limited. Second, no matter whether undergoing intravenous thrombolysis or mechanical thrombectomy, there was no statistically significant recanalization rate between the two groups. Third, studies on hemorrhagic strokes are urgent, as we know, hemorrhagic strokes are often more deadlier and devastating. By appraising SHR, earlier identification of the adverse results, such as hematoma expansion, is much vital for the neurosurgeon.

## Limitations

Some limitations to this meta-analysis are as follows: first, available studies are mainly retrospective studies other than randomized even though massive sample; second, most of the included studies were from Chinese scholars, and the articles from other countries are required. Despite these limitations, we believe that the results of our meta-analysis may be useful to the clinicians in their choice of treatment for stroke patients; third, heterogeneity in outcomes reporting is also significant due to the highly variable duration of postoperative follow-up and different SHR groupings.

## Conclusion

To our knowledge, this is the first meta-analysis assessing the association of different SHR and clinical outcomes in patients with stroke. With or without diabetes, no matter whether undergoing intravenous thrombolysis or mechanical thrombectomy, higher SHR significantly increased the occurrence of poor outcomes, mortality, neurological deficit, HT, and infectious complications. No statistically significant difference in recanalization was observed between the two groups. More attention must be paid to clinical practice. Future investigation should focus on the diagnostic value of SHR and the early control of hyperglycemia. Meanwhile, whether SHR could be a novel target for early intervention is worthy of attention in future research. Besides, the impact of the dynamic change of glucose-to-HbA1c ratio on ICH outcomes requires further investigation in future research. Although no randomized double-blind studies have been conducted, the available massive sample studies reflect the actual situation in the clinic and assist clinical decision makers.

## Data availability statement

The original contributions presented in the study are included in the article/Supplementary material, further inquiries can be directed to the corresponding author/s.

## Author contributions

Y-WH and X-SY developed the initial idea for this study, formulated the study design, and contributed to the original draft. Z-PL developed and revised the search strategy and responsible for the revision of the draft. All authors approved the final version of the manuscript before submission.

## Conflict of interest

The authors declare that the research was conducted in the absence of any commercial or financial relationships that could be construed as a potential conflict of interest.

## Publisher's note

All claims expressed in this article are solely those of the authors and do not necessarily represent those of their affiliated organizations, or those of the publisher, the editors and the reviewers. Any product that may be evaluated in this article, or claim that may be made by its manufacturer, is not guaranteed or endorsed by the publisher.
